# Urinary polycyclic aromatic hydrocarbon metabolites and their association with oxidative stress among pregnant women in Los Angeles

**DOI:** 10.1186/s12940-024-01107-w

**Published:** 2024-08-13

**Authors:** Qi Meng, Sanjali Mitra, Irish Del Rosario, Michael Jerrett, Carla Janzen, Sherin U. Devaskar, Beate Ritz

**Affiliations:** 1 Department of Epidemiology, University of California, Los Angeles, CA 90095 USA; 2 Department of Environmental Health Sciences, University of California, Los Angeles, CA 90095 USA; 3 Department of Obstetrics & Gynecology, University of California, Los Angeles, CA 90095 USA; 4 Department of Pediatrics, University of California, Los Angeles, CA 90095 USA

**Keywords:** PAH (polycyclic aromatic hydrocarbons), Oxidative stress, Pregnancy

## Abstract

**Background:**

Polycyclic aromatic hydrocarbons (PAHs) have been linked to adverse birth outcomes that have been reported to be induced by oxidative stress, but few epidemiological studies to date have evaluated associations between urinary PAH metabolites and oxidative stress biomarkers in pregnancy and identified critical periods for these outcomes and PAH exposures in pregnancy.

**Methods:**

A cohort of pregnant women was recruited early in pregnancy from antenatal clinics at the University of California Los Angeles during 2016–2019. We collected urine samples up to three times during pregnancy in a total of 159 women enrolled in the cohort. A total of 7 PAH metabolites and 2 oxidative stress biomarkers [malondialdehyde (MDA), 8-hydroxy-2’-deoxyguanosine (8-OHdG)] were measured in all available urine samples. Using multiple linear regression models, we estimated the percentage change (%) and 95% confidence interval (CI) in 8-OHdG and MDA measured at each sample collection time per doubling of PAH metabolite concentrations. Furthermore, we used linear mixed models with a random intercept for participant to estimate the associations between PAH metabolite and oxidative stress biomarker concentrations across multiple time points in pregnancy.

**Results:**

Most PAH metabolites were positively associated with both urinary oxidative stress biomarkers, MDA and 8-OHdG, with stronger associations in early and late pregnancy. A doubling of each urinary PAH metabolite concentration increased MDA concentrations by 5.8-41.1% and 8-OHdG concentrations by 13.8-49.7%. Linear mixed model results were consistent with those from linear regression models for each gestational sampling period.

**Conclusion:**

Urinary PAH metabolites are associated with increases in oxidative stress biomarkers during pregnancy, especially in early and late pregnancy.

**Supplementary Information:**

The online version contains supplementary material available at 10.1186/s12940-024-01107-w.

## Background

Polycyclic aromatic hydrocarbons (PAHs) are widespread environmental pollutants resulting from incomplete combustion or pyrolysis of organic matter [1]. Due to their ubiquity and toxicity, PAHs are of global public health concern [2]. The sources of human exposures to PAHs are diverse and include tobacco smoke, industrial sites, wildfires, residential wood fires, ambient air pollution, and dietary items such a charbroiled meat [1, 3]. Some PAHs, such as Benzo[a]pyrene and Benzo[b]fluoranthene, have been listed as carcinogens by International Agency for Research on Cancer (IARC) [4]. Moreover, PAHs can act as endocrine disrupters [5].

Prenatal exposures to PAHs have been linked to adverse birth outcomes such as fetal growth restriction and preterm birth [6, 7]. They have also been related to neurodevelopment [8] and childhood asthma [9], yet the biological mechanisms through which PAHs exhibit such toxicity are still debated. PAHs have been reported to induce oxidative stress, inflammation and endocrine disruptions, which may individually or jointly have adverse effects on the pregnancy [10–12]. Specifically, PAHs can induce an excess of reactive oxygen species (ROS), which would target DNA, proteins and lipids, resulting in their oxidation [12]. Well-established biomarkers of oxidative stress include products of oxidation processes such as malondialdehyde (MDA) and 8-hydroxy-2’-deoxyguanosine (8-OHdG), which represents lipid peroxidation and oxidative DNA damage, respectively [13].

Urinary concentrations of hydroxylated metabolites of PAHs (OH-PAHs) have been widely used as biomarkers of PAH exposure, as they represent the integrated levels of exposure across multiple pathways in the hours or days prior to sampling [14]. Nevertheless, only a few studies up to date have used urinary PAH biomarkers to examine the effect of PAH exposures on oxidative stress levels among pregnant women. A cohort study of 200 pregnant women in Boston collected urine samples in the 3rd trimester of pregnancy and found a 14% (95% CI: 0.59%, 30.1%) increase in urinary 8-hydroxy-2’-deoxyguanosine (8-OHdG) per interquartile range (IQR) increase in the PAH metabolite, 2-hydroxynapthalene (2-NAP) [15]. Similarly, several PAH metabolites including hydroxynaphthalene, hydroxyfluorene, and hydroxyphenanthrene were reported to be correlated with urinary concentrations of 8-OHdG in a cohort of 188 pregnant Chinese women (*r* = 0.3–0.6) [16]. Finally, a larger study of 715 South Korean pregnant women reported that urinary concentrations of 2-NAP (*r* = 0.26) and 1-hydroxypyrene (*r* = 0.24) were correlated with increases in MDA at 12–28 weeks of gestation [17]. Although all studies found consistent correlations between PAH metabolite and oxidative stress biomarkers, they did not collect multiple samples during pregnancy and, thus, could not control for intra-individual variability in urinary PAH metabolites or oxidative stress biomarkers across gestational periods. Some also focused on a specific time window but none could address whether critical windows exist in which PAH exposures increase oxidative stress during pregnancy.

Here, we collected urine samples from pregnant women enrolled at UCLA antenatal clinics for up to three times during pregnancy and evaluated the associations between urinary PAH metabolite concentrations and oxidative stress biomarkers in different time periods during pregnancy.

## Methods

### Study population

The Imaging Innovations for Placental Assessment in Response to Environmental Exposures (PARENTs) study recruited a cohort of 199 women early in pregnancy from antenatal clinics at the University of California Los Angeles during 2016–2019 [18]. Women were enrolled as early as the 10th week of gestation and were asked to participate in a once-per-trimester and at-birth study visit that included phone interviews to collect environmental and behavioral risk factor data, and urinary sample collections used for assessing oxidative stress biomarkers and hydroxylated metabolites of PAHs. Women were also asked to complete a web-based food frequency questionnaire (FFQ), Diet History Questionnaire II (DHQ II), in mid-pregnancy.

Our study population consists of 159 women enrolled in the PARENTs study for whom at least one useable urine sample was available at the time of laboratory analysis supported by the Emory Children’s Health Exposure Analysis Resource (CHEAR) program.

### Biomarkers assessment

We quantified two oxidative stress biomarkers, malondialdehyde (MDA) and 8-hydroxy-2’-deoxyguanosine (8-OHdG), and a total of 7 hydroxyl PAH metabolites. Specifically, we examined measures for the combined 2-hydroxyfluorene + 3-hydroxyfluorene (2&3-FLUO) metabolites, the single metabolites 2-hydroxynaphthalene (2-NAP), 1-hydroxyphenanthrene (1-PHEN), 2-hydroxyphenanthrene (2-PHEN), 3-hydroxyphenanthrene (3-PHEN), 4-hydroxyphenanthrene (4-PHEN), and 1-hydroxypyrene (1-PYR), the sum of 1-, 2-, 3-, and 4- hydroxyphenanthrene (Σ_4_OH-PHEN), and also the sum of all 7 metabolites (Σ_7_OH-PAH), respectively.

Study visits were timed to be optimal for Magnetic resonance imaging (MRI) evaluations (1st MRI 14-18 gestational weeks, 2nd MRI 19-24 gestational weeks) in the PARENTs cohort study, and sample collection followed this schedule with the 1st sample being collected in the 10-17 gestational weeks, the 2nd sample collection in the 18-29 gestational weeks, and the 3rd sample collection after the 30 gestational weeks and prior to delivery. Maternal urinary samples were collected at each study visit, and we collected at least one and at most three urine samples from all participants during pregnancy. Specifically, multiple urine samples were collected among 146 out of 159 participants (92%).

The urine samples were stored at -80 °C after collection at UCLA and were shipped on dry ice to the CHEAR Laboratory Hub to measure both the OH-PAHs and the oxidative stress biomarkers. All samples were randomized using a Fisher-Yates shuffling algorithm prior to laboratory analysis to reduce any potential batch effects [19, 20]. The OH-PAHs were measured by tandem mass spectrometry (MS/MS) [21], and the oxidative stress biomarkers (MDA and 8-OHdG) were measured by liquid chromatography-mass spectrometry (LC-MS) [22].

For both PAH metabolites and oxidative stress biomarkers, samples with measures below the limit of detection (LOD) values were replaced with the LOD/√2 [23]. Concentrations were further corrected for urine dilution by adjusting for specific gravity (SG), which was measured with a Reichert AR200 refractometer. To correct for the hydration status of pregnant women, SG-standardized biomarker concentrations were calculated using the following formula [24]:

$$\:{CHEM}_{SG\_Adj\:}={CHEM}_{i}*\left[\right({SG}_{m}-1)/({SG}_{i}-1)]\:$$

where CHEM_SG_Adj_ is the specific gravity-standardized biomarker concentration (nmol/L for MDA, ng/mL for 8-OHdG, ng/L for OH-PAHs), CHEM_i_ is the observed biomarker concentration, SG_i_ is the specific gravity of the urine sample and SG_m_ is the median specific gravity for the total samples with valid SG values. We excluded samples with an invalid SG value below or equal to 1 (*N* = 14, 18 and 16 samples during the 1st, 2nd and 3rd sample collection interval, respectively), resulting in a total of 391 samples available for analysis.

### Covariates

Potential confounders were selected a priori according to the literature considering factors that might influence oxidative stress level and maternal PAH exposures [15–17, 25]. Information on maternal age (years), parity (continuous), maternal pre-pregnancy body mass index (BMI) (< 18.5, 18.5–24.9, 25.0–29.9 and ≥ 30.0), maternal race/ethnicity (White, non-White), maternal smoking status (yes, no), maternal educational attainment (bachelor’s degree or less, master’s degree, doctoral/professional degree), and maternal employment status (employed or student, unemployed) were collected in interviews. Gestational age (based on the best obstetric estimate obtained during a 1st trimester ultrasound exam), as well as information about pregnancy complications including gestational diabetes, gestational hypertension, and pre-eclampsia were obtained from medical records. Season of sample collection was categorized based on the month of sample collection, as spring (March, April, May), summer (June, July, August), fall (September, October, November) and winter (December, January, February). The Alternate Mediterranean Diet scores (aMED) were calculated using the DHQ II data as an indicator for antioxidant intake.

### Statistical analysis

First, we estimated effects for different time intervals during pregnancy by conducting multiple linear regression analyses and calculating the expected percentage of change in each oxidative stress biomarker concentration according to PAH metabolite levels in each sample collection interval, separately. The oxidative stress biomarker concentrations were treated as continuous variables and natural log-transformed for statistical analyses. For all OH-PAHs, we log-transformed (base 2) the values such that in the statistical model the exposure effect estimate represents an increase per doubling of the OH-PAHs concentration (ng/L).

Specifically, in one-point-in-time linear regression model, the association between OH-PAHs and oxidative stress biomarkers was modeled as:

$${y_{ij}} = \alpha + \beta {x_{ij}} + \Upsilon {z_{ij}} + {\varepsilon _{ij}}$$

where y_ij_ and x_ij_ are the log-transformed concentration of each urinary oxidative stress biomarker and PAH metabolite of participant i at sampling collection period j, respectively; z_ij_ represented a set of covariates that we adjusted in the model for each participant i at sampling collection period j; α and β are the fixed intercept and slope, respectively; ϒ are the fixed slopes for the covariates; ε_ij_ is the residual.

Furthermore, we also used linear mixed models with a random intercept for each participant i to take repeated measurements (up to 3 samples across multiple time points during pregnancy) into account:

$${y_{ij}} = \alpha + {\mu _i} + \beta {x_{ij}} + \Upsilon {z_{ij}} + {\varepsilon _{ij}}$$

where µ_i_ is the random intercept for participant i.

In one-point-in-time linear and in longitudinal linear mixed regression models, we adjusted for maternal age, maternal race/ethnicity, maternal education, parity, pre-pregnancy BMI, and season of sampling. We also evaluated potential effect measure modification by season of sampling, fetal sex, and maternal race/ethnicity. Tests for heterogeneity (multiplicative scale) were performed by assessing the *p*-value of the interaction term for the exposure and the potential effect measure modifier. We also tested the heterogeneity by sampling collection period in linear mixed regression models. Sensitivity analyses were conducted by additionally adjusting for gestational day at sample collection, aMED, or maternal employment, separately. Considering the impact of multiple testing, we also adjusted the *p*-values of the multiple linear regression models in each sampling period using the False Discovery Rate (FDR) method [26]. Furthermore, as women experiencing pregnancy complications are likely to have higher oxidative stress levels due to these conditions [27], we also restricted the main analyses to women without pregnancy complications including gestational diabetes, gestational hypertension, or pre-eclampsia. All statistical analyses were performed using SAS 9.4 (SAS Institute Inc., Cary, NC, USA).

## Results

Most of our study participants were ≥ 30 years old and highly educated, more than half were parous, and almost a third (31%) were overweight or obese (BMI ≥ 25) (Table 1). Approximately half of the mothers reported their race/ethnicity as non-Hispanic White; 18% as Hispanic and 28% as Asian/Pacific Islander. The demographics characteristics of the entire PARENTs cohort are very similar to those of the subpopulation used in this study (Table S1).

**Table 1:** Characteristics of the study population (*N* = 159)

Characteristics	*N*	%
**Maternal age (years)**		
≤ 24	3	1.9
25–29	22	13.8
30–34	77	48.4
≥ 35	57	35.9
**Parity**		
0	74	46.5
≥ 1	85	53.5
**Maternal race/ethnicity**		
Asian or Pacific islander	45	28.3
Black	11	6.9
Hispanic	29	18.2
White, non-Hispanic	73	45.9
American Indian or Alaskan Native	1	0.6
**Maternal education**		
Bachelor’s degree or less	72	46.5
Master’s degree	45	29.0
Doctoral degree or professional degree	38	24.5
Missing	4	
**Employment Status**		
Employed or student	139	89.7
Not employed	16	10.3
Missing	4	
**Pre-pregnancy BMI**		
Underweight	6	3.8
Normal	103	64.8
Overweight	32	20.1
Obese	18	11.3
**Gestational Diabetes**		
Yes	20	12.6
No	139	87.4
**Gestational Hypertension**		
Yes	15	9.4
No	144	90.6
**Pre-eclampsia**		
Yes	16	10.1
No	143	89.9
**Season of Conception**		
Spring	35	22.0
Summer	42	26.4
Fall	36	22.6
Winter	46	28.9

The average MDA concentrations did not change much from the 1st to 2nd sample collection but increased in the 3rd interval (Table S2). The concentrations of 8-OHdG decreased throughout pregnancy. Most of the PAH metabolites, except for 2&3-FLUO, showed an increasing trend across the three sampling periods. For both oxidative stress biomarkers, lower concentrations were observed in summer and relatively higher concentrations in fall and/or winter (Table S3). For the PAH metabolites 2&3-FLUO, 2-NAP and sum of the OH-PHEN isomers, we measured relatively higher concentrations in summer. In each pregnancy sample collection period, the two oxidative stress biomarkers were highly correlated with each other (*r* = 0.8, 0.6, 0.8 for the 1st, 2nd and 3rd sampling, respectively), as were the OH-PHEN isomers and 1-PYR (*r* = 0.6–0.9, 0.5-0.7, 0.7-0.8 for the 1st, 2nd and 3rd sampling, respectively) (Figure S1), and the OH-PHEN isomers were moderately to highly correlated with the two oxidative stress biomarkers, except in the 2nd pregnancy sampling period (*r* = 0.4–0.8, 0.2–0.4, 0.3–0.9 for the 1st, 2nd and 3rd sampling, respectively).

**Fig. 1:**
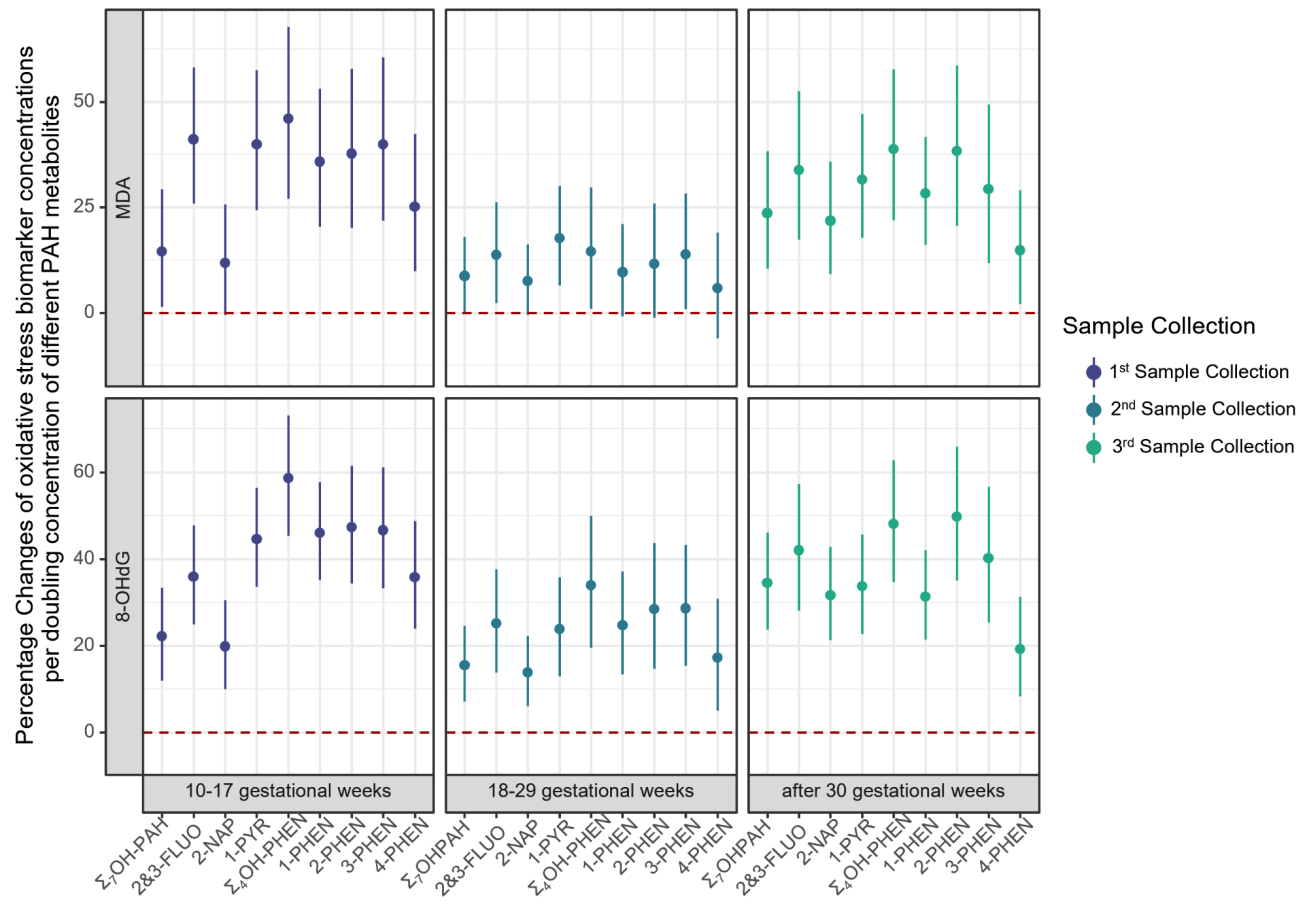
Linear regression for percentage changes of oxidative stress biomarker concentrations per doubling concentration of different PAH metabolites^a^. (a. Adjusted for maternal age, maternal race/ethnicity, maternal education, parity, pre-pregnancy BMI, and sampling season.)

In one-pregnancy period at a time linear regression models, a doubling of each urinary PAH metabolite concentration was associated with a 5.8-41.1% increase in the MDA concentrations, with the lowest effects estimated in the 2nd sampling period (Fig. 1; Figure S2; Table S4), and some of the 95% CIs including the null value. MDA concentration also increased by 8.7-23.6% in each of the three sampling periods with a doubling of the Σ_7_OH-PAH. Specifically, a doubling of 2&3-FLUO concentrations increased MDA levels by 13.7-41.1% and of 1-PYR by 17.7-39.9%. 2-NAP concentrations were associated with a 21.8% (95% CI: 9.2%, 35.8%) increase in MDA measured in the 3rd sampling period, and were also increased in the first two periods, but the 95% CIs included the null. MDA increases ranged from 14.5 to 46.0% per doubling in Σ_4_OH-PHEN; and each individual OH-PHEN isomer exhibited a similar pattern as the summary measure, with the smallest effects estimated for 4-PHEN.

Positive associations were also observed for 8-OHdG (a DNA damage marker) with each PAH exposure in simple one-pregnancy period only linear regression models. Although effect estimates were smaller in the 2nd gestational sampling period, most of the 95% CIs overlapped for the three sampling periods (Fig. 1; Figure S2; Table S4). A doubling of Σ_7_OH-PAH concentration was associated with a 22.2% (95% CI: 12.0%, 33.4%), 15.5% (95% CI: 7.1%, 24.6%) and 34.5% (95% CI: 23.7%, 46.1%) increase in urinary 8-OHdG in the 1st, 2nd and 3rd sampling period, respectively. In all three sampling periods, concentrations of 8-OHdG increased with a doubling in urinary concentrations of 2&3-FLUO by 25.1-42.0%, 2-NAP by 13.8-31.6%, and 1-PYR by 23.8-44.6%, respectively. Σ_4_OH-PHEN were also found to increase 8-OHdG in all three sampling periods by 33.9-58.6%; and similar to MDA, smaller effect estimates were observed for 4-PHEN.

**Fig. 2:**
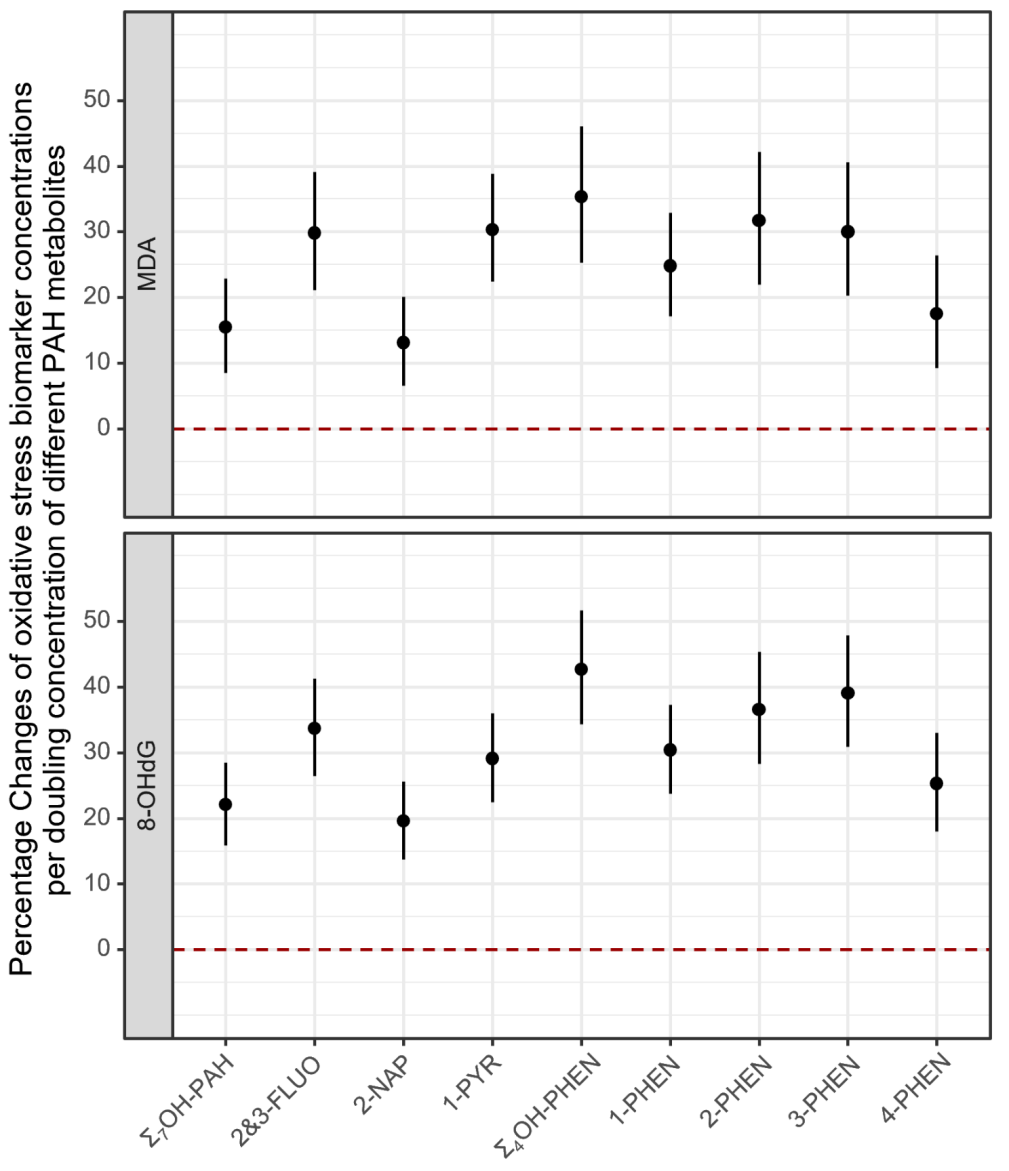
Linear mixed model for percentage changes of oxidative stress biomarker concentrations per doubling concentration of different PAH metabolites^a^ (a. Adjusted for maternal age, maternal race/ethnicity, maternal education, parity, pre-pregnancy BMI, and sampling season.)

Linear mixed models that included all three pregnancy sample collection periods showed consistent patterns with those from separate linear regression models (Fig. 2; Table S5). We estimated a 15.5% (95% CI: 8.5%, 22.9%) increase of MDA levels and a 22.1% (95% CI: 15.9%, 28.5%) increase of 8-OHdG levels associated with a doubling of Σ_7_OH-PAH concentration. The effect estimates for specific PAH metabolites ranged from 13.1 to 31.7% for MDA concentrations, and from 19.6 to 39.1% for 8-OHdG concentrations, with somewhat less strong associations for 2-NAP and 4-PHEN. The FDR adjusted *p*-values were still statistically significant at a significance level of 0.05 (Table S6). When testing the heterogeneity of sampling collection period in the linear mixed models, we estimated slightly higher increases in both MDA and 8-OHdG concentrations per doubling of most of the PAH metabolite concentrations, yet the 95% CIs widened and mostly overlapped with those from the main model (data not shown). The interaction *p*-values were statistically significant (< 0.05) for the 2nd sampling period compared with the 3rd sampling period as reference (Table S4).

When stratifying by sampling season, in both simple one-pregnancy sampling period linear regression models (data not shown) and linear mixed regression models, stronger effect estimates were generally observed in fall and winter for most PAH exposures and both oxidative stress biomarkers (Figure S3). We did not observe any heterogeneity by fetal sex or maternal race/ethnicity in both one-point-in-time linear regression (data not shown) or the linear mixed regression models (Figure S4 and Figure S5).

Finally, in one-pregnancy-time-point linear regression, effect estimates slightly increased after restricting to women without pregnancy complications (Table S7). Results changed minimally after additionally adjusting for gestational days at sample collection date, aMED, or maternal employment status (data not shown).

## Discussion

In our study of pregnant women, we found consistent associations between several urinary PAH metabolites and two oxidative stress biomarkers for lipid peroxidation (MDA), and DNA damage (8-OHdG). Stronger associations were seen in early and late pregnancy, especially for urine samples collected in the fall or winter season. To our knowledge, this is the first longitudinal study with repeated urinary measures to assess associations between urinary PAH metabolites and two oxidative stress biomarkers during pregnancy. This also allowed us to additionally investigate whether there are susceptible windows for PAH exposures across pregnancy.

The metabolism of PAHs depends on the cytochrome P450-mediated mixed function oxidase system that produces enormous quantities of reactive intermediates involved in lipid peroxidation, protein modification, DNA damage, and the depletion of endogenous antioxidants [28]. Our findings for PAH metabolites including 2&3-FLUO, 2-NAP, 1-PYR, and OH-PHENs being associated with increases in urinary 8-OHdG and MDA during pregnancy are consistent with the few previously published reports investigating this subject [15–17]. However, studies in pregnant women that investigate associations between PAH exposures and oxidative stress in different time periods during pregnancy are rare. The stronger associations we observed in early and late pregnancy were consistent with the sensitive window for adverse birth outcomes such as preterm birth and term low birth weight, which have been widely reported to be associated with prenatal exposures to environmental pollutants and the oxidative stress they induce [29–32].

Stronger associations between PAH exposures and oxidative stress biomarkers have also been detected in the winter season. This may be explained by seasonally higher atmospheric PAH concentrations in winter than summer for most compounds due to an increased production from heating related combustion and adverse meteorological conditions [33]. It may also suggest higher toxicity of the mixture from combustion sources. Apart from smoking and ambient or indoor air pollution from traffic and combustion including open fireplaces and wood burning, dietary intake is one of the main sources of PAH exposures in the general population. This includes food contamination and food processing procedures such as smoking, drying, and frying of foods [28], which makes diet a potential confounder of the association between PAH exposures and oxidative stress. In our study, we adjusted for aMED as an indicator for antioxidant intake and found that our results were robust. Maternal employment conditions may also confound the associations, as work-related stress was reported to be associated with urinary concentrations of 8-OHdG in female workers [34, 35]. Our results, however, were robust after adjustment for maternal employment status, possibly because most of the study participants were students or employees of UCLA, i.e., there is likely not much variation in work-related exposure resulting in oxidative stress.

Oxidative stress is one of the main mechanisms hypothesized to lead to suboptimal placentation and in turn affect pregnancy success [36]. Our previous study found oxidative stress from air pollution to be associated with adverse birth outcomes [37] as well as with oxidative stress biomarker concentrations in pregnant women [38]. Previous studies have also linked ambient PAH pollutants with adverse birth outcomes [39, 40]. A study of 1,677 women measured urinary PAH metabolites in the 2nd trimester and linked these to preterm birth [7], and a recent study also reported associations between urinary OH-PAH concentrations in mid-pregnancy and the placental transcriptome [41].

Oxidative stress has also emerged as one of the underlying mechanism contributing to the toxicity of endocrine disrupting chemicals [42]. In addition, hormonal imbalance leads to compromised antioxidant status and oxidative stress in tissues induces various pathophysiological conditions [43]. Thus, altered hormonal signaling by PAH exposures may contribute to sex differences in oxidative stress levels. A recent cohort study of pregnant women has observed the endocrine disrupting potential of PAH exposures with sex-specific changes in hormone concentrations in pregnancy, which can result in adverse birth outcomes [10, 44]. Oxidative stress pathways may mediate the impact of PAH exposures on adverse birth outcomes via these biological pathways. We however did not observe formally statistically significant sex differences possibly due to our limited sample size. Further studies are necessary to document and understand placental sex differences in response to PAH exposures.

Our study has several strengths. First, the repeated measures in different pregnancy periods made it possible to investigate how the PAH metabolite concentrations throughout pregnancy influence oxidative stress. In addition, using repeated urinary measures in linear mixed models also made it possible to account for intra-individual variability in oxidative stress biomarkers. Second, the detailed data we collected for environmental and medical covariates enabled sensitivity analyses, such as excluding women more likely to exhibit higher oxidative stress levels such as due to pregnancy disorders. Third, our study population is highly educated and mostly of high socioeconomic status, such that occupational exposures, which are often higher than environmental exposures, are unlikely to have played a role as confounders [45]. Lastly, we used two different oxidative stress biomarkers and evaluated both DNA oxidative damage and lipid peroxidation.

Some limitations need to also be acknowledged. First, the short biological half-lives of PAHs limits the informativeness of urinary PAH metabolites as they only reflect recent exposures and to imply that they represent longer term exposures we have to assume relatively constancy of exposures over time [12, 46]. We analyzed urine samples for both the exposure and the outcomes, thus technically using a multiple time point cross-sectional design. Nevertheless, reverse-causation is likely not a concern as PAHs are environmental toxicants that the body metabolizes but does not produce and oxidative stress is a likely consequence, not cause of PAH exposures. Second, some studies have suggested that chronic exposures to PAHs may contribute to lower renal function, and lower maternal glomerular filtration rates may affect both the excretion of PAH metabolites and of oxidative stress biomarkers [47, 48]. Women with preexisting renal function impairment at the time of sample collection may have exhibited altered excretion of both PAH metabolites and oxidative stress biomarkers resulting in residual confounding. However, in sensitivity analysis that excluded women with pregnancy complications we likely excluded women with renal function impairment and estimated effects were consistent with the results we presented. Furthermore, even though we collected up to three samples in pregnancy for the participants, our sample size was limited, especially for subgroup analyses. Larger prospective studies with multiple sample collection time points may help better understand these mechanisms.

## Conclusion

We found that PAH exposures measured with urinary PAH metabolites are associated with increased oxidative stress generation during pregnancy, which has been previously linked to adverse birth outcomes.

### Electronic supplementary material

Below is the link to the electronic supplementary material.

Supplementary Material 1

## Data Availability

Data required to reproduce the above findings will be shared via the Human Health Exposure Analysis Resource [HHEAR, formerly Children’s Health Exposure Analysis Resource (CHEAR)] platform maintained by NIEHS (National Institute of Environmental Health Sciences of the National Institutes of Health) contractors.

## Abbreviations

PAH: Polycyclic aromatic hydrocarbons
IARC: International Agency for Research on Cancer
ROS: Reactive oxygen species
8-OHdG: 8-hydroxy-2’-deoxyguanosine
MDA: Malondialdehyde
OH-PAHs: Hydroxylated metabolites of polycyclic aromatic hydrocarbons
2-NAP: 2-hydroxynapthalene
PARENTs: Imaging Innovations for Placental Assessment in Response to Environmental Exposures study
CHEAR: Children’s Health Exposure Analysis Resource
2&3-FLUO: Combined 2-hydroxyfluorene + 3-hydroxyfluorene metabolites
1-PHEN: 1-hydroxyphenanthrene
2-PHEN: 2-hydroxyphenanthrene
3-PHEN: 3-hydroxyphenanthrene
4-PHEN: 4-hydroxyphenanthrene
1-PYR: 1-hydroxypyrene
Σ4OH-PHEN: Sum of 1-, 2-, 3-, and 4- hydroxyphenanthrene
Σ7OH-PAH: Sum of combined 2-hydroxyfluorene + 3-hydroxyfluorene metabolites, 2-hydroxynapthalene, 1-hydroxypyrene, 1-hydroxyphenanthrene, 2-hydroxyphenanthrene, 3-hydroxyphenanthrene, and 4-hydroxyphenanthrene
MRI: Magnetic resonance imaging
LOD: Limit of detection
SG: Specific gravity
BMI: Body mass index
aMED: Alternate Mediterranean Diet scores
FDR: False Discovery Rate
