# Supplementary material for: Urinary polycyclic aromatic hydrocarbon metabolites and their association with oxidative stress among pregnant women in Los Angeles
Source: Environ Health. 2024 Aug 13;23:68. doi: 10.1186/s12940-024-01107-w (PMC11321171; doi:10.1186/s12940-024-01107-w)

**Supplementary Materials**

| **Table S1 Characteristics of the PARENTs cohort (N=199)** | | |
| --- | --- | --- |
| **Characteristics** | **N** | **%** |
| **Maternal age (years)** |  |  |
| ≤24 | 4 | 2.0 |
| 25-29 | 29 | 14.6 |
| 30-34 | 92 | 46.2 |
| ≥35 | 74 | 37.2 |
| **Parity** |  |  |
| 0 | 94 | 47.2 |
| ≥1 | 105 | 52.8 |
| **Maternal race/ethnicity** |  |  |
| Asian or Pacific islander | 52 | 26.1 |
| Black | 14 | 7.0 |
| Hispanic | 40 | 20.1 |
| White, non-Hispanic | 92 | 46.2 |
| American Indian or Alaskan Native | 1 | 0.5 |
| **Maternal education** |  |  |
| Bachelor's degree or less | 84 | 45.2 |
| Master's degree | 57 | 30.7 |
| Doctoral degree or professional degree | 45 | 24.2 |
| Missing | 13 |  |
| **Employment Status** |  |  |
| Employed or student | 167 | 90.3 |
| Not employed | 18 | 9.7 |
| Missing | 14 |  |
| **Pre-pregnancy BMI** |  |  |
| Underweight | 6 | 3.0 |
| Normal | 120 | 60.3 |
| Overweight | 49 | 24.6 |
| Obese | 24 | 12.1 |
| **Gestational Diabetes** |  |  |
| Yes | 22 | 11.1 |
| No | 177 | 88.9 |
| **Gestational Hypertension** |  |  |
| Yes | 22 | 11.1 |
| No | 177 | 88.9 |
| **Pre-eclampsia** |  |  |
| Yes | 18 | 9.1 |
| No | 181 | 91.0 |
| **Season of Conception** |  |  |
| Spring | 45 | 23.3 |
| Summer | 47 | 24.4 |
| Fall | 42 | 21.8 |
| Winter | 59 | 30.6 |
| Missing | 6 |  |

**Table S2 Specific Gravity (SG) adjusted concentrations of urinary biomarkers in each sample collection**

| **Biomarkers** | **N** | **Specific Gravity (SG) adjusted Value ^a^** | | | | | |
| --- | --- | --- | --- | --- | --- | --- | --- |
|  |  | **Minimum** | **Maximum** | **Geometric Mean** | **25th Pct** | **50th Pct** | **75th Pct** |
| **1st sample collection (10-17 gestational weeks)** | | | | | | | |
| **MDA (nmol/L)** | 139 | 9.4 | 2370.0 | 184.5 | 119.0 | 190.0 | 281.0 |
| **8-OHdG (µg/L)** | 136 | 0.4 | 34.0 | 2.7 | 1.9 | 2.6 | 3.8 |
| **Σ_7_OHPAH (ng/L)** | 138 | 862.7 | 38748.3 | 4136.1 | 2203.5 | 3460.2 | 6307.2 |
| **2- & 3-FLUO (ng/L)** | 139 | 10.4 | 2340.0 | 107.7 | 62.0 | 95.7 | 175.4 |
| **2-NAP (ng/L)** | 139 | 768.6 | 37100.0 | 3719.7 | 1964.2 | 2946.3 | 5892.6 |
| **1-PYR (ng/L)** | 139 | 5.2 | 1430.0 | 42.3 | 24.7 | 37.8 | 68.3 |
| **Σ_4_OHPHEN (ng/L)** | 138 | 26.0 | 2265.5 | 140.1 | 88.4 | 130.9 | 206.9 |
| **1-PHEN (ng/L)** | 139 | 5.5 | 1665.0 | 63.5 | 38.0 | 63.5 | 99.3 |
| **2-PHEN (ng/L)** | 138 | 5.2 | 300.0 | 26.1 | 16.3 | 24.3 | 39.3 |
| **3-PHEN (ng/L)** | 138 | 5.2 | 253.0 | 27.1 | 16.0 | 25.2 | 40.0 |
| **4-PHEN (ng/L)** | 139 | 3.4 | 241.0 | 16.3 | 9.8 | 14.7 | 24.0 |
| **2nd sample collection (18-29 gestational weeks)** | | | | | | | |
| **MDA (nmol/L)** | 137 | 33.0 | 1205 | 185.0 | 119.4 | 167.9 | 274.4 |
| **8-OHdG (µg/L)** | 134 | 0.2 | 18.1 | 2.4 | 1.6 | 2.2 | 3.0 |
| **Σ_7_OHPAH (ng/L)** | 137 | 895.6 | 114824.5 | 4463.2 | 2217.2 | 3894.4 | 8097.7 |
| **2- & 3-FLUO (ng/L)** | 137 | 18.1 | 1146.0 | 117.0 | 71.3 | 112.1 | 174.4 |
| **2-NAP (ng/L)** | 137 | 631.3 | 114545.5 | 4009.8 | 1964.2 | 3535.5 | 7473.7 |
| **1-PYR (ng/L)** | 137 | 9.8 | 746.7 | 48.7 | 29.4 | 45.0 | 63.9 |
| **Σ_4_OHPHEN (ng/L)** | 137 | 40.4 | 841.0 | 149.2 | 103.8 | 140.2 | 209.6 |
| **1-PHEN (ng/L)** | 137 | 5.2 | 450.0 | 72.5 | 49.1 | 69.6 | 113.0 |
| **2-PHEN (ng/L)** | 137 | 4.7 | 156.7 | 27.7 | 19.1 | 26.4 | 34.9 |
| **3-PHEN (ng/L)** | 137 | 4.9 | 133.0 | 25.6 | 17.5 | 24.0 | 37.4 |
| **4-PHEN (ng/L)** | 137 | 3.3 | 135.4 | 16.3 | 10.8 | 17.0 | 22.1 |
| **3rd sample collection (≥30 gestational weeks)** | | | | | | | |
| **MDA (nmol/L)** | 115 | 12.7 | 5110.0 | 193.5 | 129.1 | 167.0 | 285.0 |
| **8-OHdG (µg/L)** | 108 | 0.2 | 22.7 | 1.9 | 1.5 | 1.9 | 2.3 |
| **Σ_7_OHPAH (ng/L)** | 115 | 895.0 | 265794.4 | 5012.3 | 2728.6 | 4311.6 | 6571.9 |
| **2- & 3-FLUO (ng/L)** | 115 | 17.7 | 2690.0 | 105.3 | 58.9 | 92.2 | 183.0 |
| **2-NAP (ng/L)** | 115 | 679.9 | 262000.0 | 4531.6 | 2495.2 | 3828.6 | 6166.7 |
| **1-PYR (ng/L)** | 115 | 8.0 | 2250.0 | 61.9 | 36.3 | 54.1 | 95.5 |
| **Σ_4_OHPHEN (ng/L)** | 115 | 18.6 | 2423.4 | 173.8 | 101.7 | 152.1 | 264.0 |
| **1-PHEN (ng/L)** | 115 | 4.7 | 1860.0 | 85.1 | 46.6 | 81.4 | 159.2 |
| **2-PHEN (ng/L)** | 115 | 4.7 | 496.7 | 33.1 | 20.7 | 28.3 | 51.9 |
| **3-PHEN (ng/L)** | 115 | 4.7 | 376.7 | 25.6 | 14.7 | 23.2 | 38.5 |
| **4-PHEN (ng/L)** | 115 | 2.9 | 225.7 | 16.0 | 8.8 | 14.7 | 29.5 |
| a. Concentrations below the limit of detection (LOD) values were replaced with the LOD/√2 | | | | | | | |

**Table S3 Specific Gravity (SG) adjusted concentrations of urinary biomarkers by sample collection season**

| **Biomarkers** | **N** | **Specific Gravity (SG) adjusted Value ^a^** | | | | | |
| --- | --- | --- | --- | --- | --- | --- | --- |
|  |  | **Minimum** | **Maximum** | **Geometric mean** | **25^th^ Pct** | **50^th^ Pct** | **75^th^ Pct** |
| **Spring** | | | | | | | |
| **MDA (nmol/L)** | 120 | 33.0 | 1500.0 | 185.1 | 117.3 | 185.7 | 275.6 |
| **8-OHdG (µg/L)** | 116 | 0.3 | 24.5 | 2.5 | 1.7 | 2.2 | 3.5 |
| **Σ_7_OHPAH (ng/L)** | 120 | 1056.4 | 89760.4 | 4238.0 | 2384.3 | 3881.6 | 6098.8 |
| **2- & 3-FLUO (ng/L)** | 120 | 18.1 | 880.0 | 101.3 | 61.4 | 88.6 | 157.7 |
| **2-NAP (ng/L)** | 120 | 841.8 | 89523.8 | 3838.8 | 1964.2 | 3535.5 | 5890 |
| **1-PYR (ng/L)** | 120 | 9.8 | 1430.0 | 48.6 | 29.4 | 44.3 | 76.1 |
| **Σ_4_OHPHEN (ng/L)** | 120 | 18.6 | 2265.5 | 145.3 | 94.5 | 135.2 | 217.4 |
| **1-PHEN (ng/L)** | 120 | 4.7 | 1665.0 | 68.1 | 37.7 | 71.7 | 109.0 |
| **2-PHEN (ng/L)** | 120 | 4.7 | 229.0 | 27.8 | 18.5 | 27.4 | 36.7 |
| **3-PHEN (ng/L)** | 120 | 4.7 | 200.5 | 25.0 | 15.1 | 22.1 | 37.4 |
| **4-PHEN (ng/L)** | 120 | 2.9 | 197.0 | 15.6 | 9.8 | 14.7 | 22.8 |
| **Summer** | | | | | | | |
| **MDA (nmol/L)** | 89 | 12.7 | 908.9 | 180.1 | 126.0 | 178.5 | 292.2 |
| **8-OHdG (µg/L)** | 85 | 0.4 | 6.8 | 2.1 | 1.6 | 2.1 | 2.7 |
| **Σ_7_OHPAH (ng/L)** | 89 | 895.6 | 33779.7 | 4865.1 | 2168.9 | 4300.9 | 9471.1 |
| **2- & 3-FLUO (ng/L)** | 89 | 10.4 | 2340.0 | 120.0 | 78.2 | 112.0 | 173.3 |
| **2-NAP (ng/L)** | 89 | 631.3 | 33450.0 | 4360.2 | 1964.2 | 3863.2 | 8838.8 |
| **1-PYR (ng/L)** | 89 | 9.2 | 746.7 | 50.0 | 29.0 | 50.4 | 79.0 |
| **Σ_4_OHPHEN (ng/L)** | 89 | 29.1 | 1362.2 | 161.1 | 98.9 | 148.6 | 216.1 |
| **1-PHEN (ng/L)** | 89 | 5.5 | 868.0 | 75.7 | 43.5 | 67.2 | 118.3 |
| **2-PHEN (ng/L)** | 89 | 5.5 | 288.1 | 29.3 | 18.5 | 25.4 | 41.4 |
| **3-PHEN (ng/L)** | 89 | 5.2 | 193.6 | 27.5 | 17.7 | 25.4 | 41.7 |
| **4-PHEN (ng/L)** | 89 | 3.3 | 135.4 | 15.9 | 9.8 | 14.7 | 25.5 |
| **Fall** | | | | | | | |
| **MDA (nmol/L)** | 88 | 59.6 | 5110.0 | 212.9 | 136.3 | 193 | 278.1 |
| **8-OHdG (µg/L)** | 85 | 0.8 | 20.4 | 2.4 | 1.6 | 2.2 | 2.9 |
| **Σ_7_OHPAH (ng/L)** | 87 | 895.0 | 265794.4 | 4357.5 | 2350.0 | 3697.4 | 6340.4 |
| **2- & 3-FLUO (ng/L)** | 88 | 17.7 | 837.0 | 112.1 | 64.5 | 106.4 | 188.6 |
| **2-NAP (ng/L)** | 88 | 679.9 | 262000.0 | 3905.2 | 2199.3 | 3527.8 | 5755.8 |
| **1-PYR (ng/L)** | 88 | 8.0 | 2250.0 | 50.7 | 30.9 | 46.0 | 73.8 |
| **Σ_4_OHPHEN (ng/L)** | 87 | 39.8 | 2423.4 | 156.5 | 102.7 | 142.9 | 213.4 |
| **1-PHEN (ng/L)** | 88 | 14.5 | 1860.0 | 77.7 | 50.2 | 75.1 | 116.5 |
| **2-PHEN (ng/L)** | 87 | 4.7 | 268.0 | 28.7 | 19.1 | 26.7 | 43.7 |
| **3-PHEN (ng/L)** | 87 | 8.2 | 207.0 | 25.6 | 16.5 | 23.3 | 36.3 |
| **4-PHEN (ng/L)** | 88 | 3.7 | 205.0 | 16.2 | 11.0 | 16.8 | 22.3 |
| **Winter** | | | | | | | |
| **MDA (nmol/L)** | 94 | 9.4 | 2370.0 | 175.0 | 103.2 | 164.4 | 274.4 |
| **8-OHdG (µg/L)** | 92 | 0.2 | 34.0 | 2.3 | 1.6 | 2.2 | 3.2 |
| **Σ_7_OHPAH (ng/L)** | 94 | 862.7 | 114824.5 | 4630.7 | 2287.1 | 3590.1 | 7260.2 |
| **2- & 3-FLUO (ng/L)** | 94 | 10.4 | 2690.0 | 111.1 | 61.1 | 110.8 | 183.0 |
| **2-NAP (ng/L)** | 94 | 768.6 | 114545.5 | 4172.2 | 1987.5 | 3293.9 | 7027.3 |
| **1-PYR (ng/L)** | 94 | 5.2 | 1130.0 | 49.9 | 29.5 | 43.5 | 78.6 |
| **Σ_4_OHPHEN (ng/L)** | 94 | 26.0 | 2382.3 | 150.8 | 89.7 | 131.3 | 224.5 |
| **1-PHEN (ng/L)** | 94 | 5.9 | 1283.3 | 70.7 | 44.3 | 66.6 | 111.0 |
| **2-PHEN (ng/L)** | 94 | 4.9 | 496.7 | 28.8 | 18.4 | 25.8 | 42.1 |
| **3-PHEN (ng/L)** | 94 | 4.9 | 376.7 | 26.8 | 17.1 | 24.6 | 41.2 |
| **4-PHEN (ng/L)** | 94 | 3.3 | 241.0 | 17.3 | 10.0 | 17.1 | 28.1 |
| a. Concentrations below the limit of detection (LOD) values were replaced with the LOD/√2 | | | | | | | |

**Table S4 Linear regression for percentage changes and 95% confidence intervals (CI) in oxidative stress biomarker concentrations per doubling concentration of different PAH metabolites**

| **Oxidative stress biomarkers** | **PAH metabolites** | **Percentage changes (95% CI) ^a^** | | | | | |
| --- | --- | --- | --- | --- | --- | --- | --- |
|  |  | **1^st^ Sampling** | **Interaction *p*-value** | **2^nd^ Sampling** | **Interaction *p*-value** | **3^rd^ Sampling** | **Interaction *p*-value** |
| **MDA (nmol/L)** | **Σ_7_OHPAH (ng/L)** | 14.5 (1.4, 29.3) | 0.36 | 8.7 (0.2, 18.0) | 0.06 | 23.6 (10.5, 38.3) | ref |
|  | **2- & 3-FLUO (ng/L)** | 41.1 (25.9, 58.2) | 0.89 | 13.7 (2.3, 26.3) | 0.03 | 33.8 (17.3, 52.6) | ref |
|  | **2-NAP (ng/L)** | 11.8 (-0.5, 25.7) | 0.27 | 7.5 (-0.5, 16.2) | 0.06 | 21.8 (9.2, 35.8) | ref |
|  | **1-PYR (ng/L)** | 39.9 (24.3, 57.5) | 0.58 | 17.7 (6.5, 30.1) | 0.14 | 31.6 (17.8, 47.1) | ref |
|  | **Σ_4_OHPHEN (ng/L)** | 46.0 (27.0, 67.8) | 0.85 | 14.5 (1.0, 29.8) | 0.02 | 38.8 (22.0, 57.8) | ref |
|  | **1-PHEN (ng/L)** | 35.8 (20.4, 53.1) | 0.84 | 9.6 (-0.8, 21.1) | 0.02 | 28.3 (16.1, 41.7) | ref |
|  | **2-PHEN (ng/L)** | 37.7 (20.2, 57.9) | 0.69 | 11.6 (-1.2, 25.9) | 0.02 | 38.3 (20.6, 58.7) | ref |
|  | **3-PHEN (ng/L)** | 39.9 (21.8, 60.6) | 0.55 | 13.8 (0.9, 28.3) | 0.15 | 29.3 (11.8, 49.4) | ref |
|  | **4-PHEN (ng/L)** | 25.1 (9.9, 42.4) | 0.43 | 5.8 (-6.0, 19.0) | 0.49 | 14.8 (2.1, 29.1) | ref |
| **8-OHdG (µg/L)** | **Σ_7_OHPAH (ng/L)** | 22.2 (12.0, 33.4) | 0.11 | 15.5 (7.1, 24.6) | 0.02 | 34.5 (23.7, 46.1) | ref |
|  | **2- & 3-FLUO (ng/L)** | 35.9 (24.9, 47.8) | 0.25 | 25.1 (13.8, 37.6) | 0.04 | 42.0 (28.1, 57.3) | ref |
|  | **2-NAP (ng/L)** | 19.8 (10.0, 30.5) | 0.10 | 13.8 (6.0, 22.3) | 0.02 | 31.6 (21.3, 42.8) | ref |
|  | **1-PYR (ng/L)** | 44.6 (33.6, 56.4) | 0.38 | 23.8 (12.9, 35.8) | 0.15 | 33.7 (22.7, 45.7) | ref |
|  | **Σ_4_OHPHEN (ng/L)** | 58.6 (45.3, 73.1) | 0.80 | 33.9 (19.5, 50.0) | 0.11 | 48.1 (34.7, 62.8) | ref |
|  | **1-PHEN (ng/L)** | 46.0 (35.1, 57.8) | 0.41 | 24.7 (13.4, 37.1) | 0.31 | 31.3 (21.4, 42.0) | ref |
|  | **2-PHEN (ng/L)** | 47.3 (34.4, 61.5) | 0.38 | 28.4 (14.7, 43.7) | 0.04 | 49.7 (35.0, 65.9) | ref |
|  | **3-PHEN (ng/L)** | 46.6 (33.3, 61.2) | 0.84 | 28.6 (15.4, 43.3) | 0.09 | 40.2 (25.4, 56.6) | ref |
|  | **4-PHEN (ng/L)** | 35.8 (23.9, 48.7) | 0.17 | 17.2 (5.0, 30.8) | 0.48 | 19.2 (8.3, 31.3) | ref |
| a. Adjusted for maternal age, maternal race/ethnicity, maternal education, parity, pre-pregnancy BMI, season of sampling. | | | | | | | |

**Table S5 Linear mixed regression models for percentage changes and 95% confidence intervals (CI) in oxidative stress biomarker concentrations per doubling concentration of different PAH metabolites**

| **Oxidative stress biomarkers** | **PAH metabolites** | **Percentage changes (95% CI) ^a^** |
| --- | --- | --- |
| **MDA (nmol/L)** | **Σ_7_OHPAH (ng/L)** | 15.5 (8.5, 22.9) |
|  | **2- & 3-FLUO (ng/L)** | 29.8 (21.1, 39.1) |
|  | **2-NAP (ng/L)** | 13.1 (6.6, 20.1) |
|  | **1-PYR (ng/L)** | 30.3 (22.4, 38.8) |
|  | **Σ_4_OHPHEN (ng/L)** | 35.3 (25.3, 46.1) |
|  | **1-PHEN (ng/L)** | 24.8 (17.1, 32.9) |
|  | **2-PHEN (ng/L)** | 31.7 (22.0, 42.2) |
|  | **3-PHEN (ng/L)** | 30.0 (20.3, 40.6) |
|  | **4-PHEN (ng/L)** | 17.5 (9.3, 26.4) |
| **8-OHdG (µg/L)** | **Σ_7_OHPAH (ng/L)** | 22.1 (15.9, 28.5) |
|  | **2- & 3-FLUO (ng/L)** | 33.7 (26.5, 41.3) |
|  | **2-NAP (ng/L)** | 19.6 (13.8, 25.6) |
|  | **1-PYR (ng/L)** | 29.1 (22.5, 36.0) |
|  | **Σ_4_OHPHEN (ng/L)** | 42.7 (34.3, 51.7) |
|  | **1-PHEN (ng/L)** | 30.4 (23.8, 37.3) |
|  | **2-PHEN (ng/L)** | 36.6 (28.3, 45.4) |
|  | **3-PHEN (ng/L)** | 39.1 (30.9, 47.9) |
|  | **4-PHEN (ng/L)** | 25.3 (18.0, 33.0) |
| a. Adjusted for maternal age, maternal race/ethnicity, maternal education, parity, pre-pregnancy BMI, season of sampling. | | |

**Table S6 Linear regression for percentage changes and 95% confidence intervals (CI) in oxidative stress biomarker concentrations per doubling concentration of different PAH metabolites, with False Discovery Rate (FDR) adjusted p-values**

| **Oxidative stress biomarkers** | **PAH metabolites** | **Percentage changes (95% CI) ^a^** | | | | | |
| --- | --- | --- | --- | --- | --- | --- | --- |
|  |  | **1^st^ Sampling** | **FDR adjusted p-values** | **2^nd^ Sampling** | **FDR adjusted p-values** | **3^rd^ Sampling** | **FDR adjusted p-values** |
| **MDA (nmol/L)** | **Σ_7_OHPAH (ng/L)** | 14.5 (1.4, 29.3) | 0.03 | 8.7 (0.2, 18.0) | 0.06 | 23.6 (10.5, 38.3) | 0.00 |
|  | **2- & 3-FLUO (ng/L)** | 41.1 (25.9, 58.2) | <.0001 | 13.7 (2.3, 26.3) | 0.03 | 33.8 (17.3, 52.6) | <.0001 |
|  | **2-NAP (ng/L)** | 11.8 (-0.5, 25.7) | 0.06 | 7.5 (-0.5, 16.2) | 0.08 | 21.8 (9.2, 35.8) | 0.00 |
|  | **1-PYR (ng/L)** | 39.9 (24.3, 57.5) | <.0001 | 17.7 (6.5, 30.1) | 0.00 | 31.6 (17.8, 47.1) | <.0001 |
|  | **Σ_4_OHPHEN (ng/L)** | 46.0 (27.0, 67.8) | <.0001 | 14.5 (1.0, 29.8) | 0.05 | 38.8 (22.0, 57.8) | <.0001 |
|  | **1-PHEN (ng/L)** | 35.8 (20.4, 53.1) | <.0001 | 9.6 (-0.8, 21.1) | 0.08 | 28.3 (16.1, 41.7) | <.0001 |
|  | **2-PHEN (ng/L)** | 37.7 (20.2, 57.9) | <.0001 | 11.6 (-1.2, 25.9) | 0.08 | 38.3 (20.6, 58.7) | <.0001 |
|  | **3-PHEN (ng/L)** | 39.9 (21.8, 60.6) | <.0001 | 13.8 (0.9, 28.3) | 0.05 | 29.3 (11.8, 49.4) | 0.00 |
|  | **4-PHEN (ng/L)** | 25.1 (9.9, 42.4) | 0.00 | 5.8 (-6.0, 19.0) | 0.35 | 14.8 (2.1, 29.1) | 0.02 |
| **8-OHdG (µg/L)** | **Σ_7_OHPAH (ng/L)** | 22.2 (12.0, 33.4) | <.0001 | 15.5 (7.1, 24.6) | 0.00 | 34.5 (23.7, 46.1) | <.0001 |
|  | **2- & 3-FLUO (ng/L)** | 35.9 (24.9, 47.8) | <.0001 | 25.1 (13.8, 37.6) | <.0001 | 42.0 (28.1, 57.3) | <.0001 |
|  | **2-NAP (ng/L)** | 19.8 (10.0, 30.5) | <.0001 | 13.8 (6.0, 22.3) | 0.00 | 31.6 (21.3, 42.8) | <.0001 |
|  | **1-PYR (ng/L)** | 44.6 (33.6, 56.4) | <.0001 | 23.8 (12.9, 35.8) | <.0001 | 33.7 (22.7, 45.7) | <.0001 |
|  | **Σ_4_OHPHEN (ng/L)** | 58.6 (45.3, 73.1) | <.0001 | 33.9 (19.5, 50.0) | <.0001 | 48.1 (34.7, 62.8) | <.0001 |
|  | **1-PHEN (ng/L)** | 46.0 (35.1, 57.8) | <.0001 | 24.7 (13.4, 37.1) | <.0001 | 31.3 (21.4, 42.0) | <.0001 |
|  | **2-PHEN (ng/L)** | 47.3 (34.4, 61.5) | <.0001 | 28.4 (14.7, 43.7) | <.0001 | 49.7 (35.0, 65.9) | <.0001 |
|  | **3-PHEN (ng/L)** | 46.6 (33.3, 61.2) | <.0001 | 28.6 (15.4, 43.3) | <.0001 | 40.2 (25.4, 56.6) | <.0001 |
|  | **4-PHEN (ng/L)** | 35.8 (23.9, 48.7) | <.0001 | 17.2 (5.0, 30.8) | 0.01 | 19.2 (8.3, 31.3) | 0.00 |
| a. Adjusted for maternal age, maternal race/ethnicity, maternal education, parity, pre-pregnancy BMI, season of sampling. | | | | | | | |

**Table S7 Linear regression for percentage changes and 95% confidence intervals (CI) in oxidative stress biomarker concentrations per doubling concentration of different PAH biomarkers among women without pregnancy complications ^a^**

| **Oxidative stress biomarkers** | **PAH metabolites** | **1^st^ Sampling** | **2^nd^ Sampling** | **3^rd^ Sampling** |
| --- | --- | --- | --- | --- |
| **MDA (nmol/L)** | **Σ_7_OHPAH (ng/L)** | 9.6 (-4.2, 25.2) | 14.5 (3.3, 26.8) | 27.3 (12.8, 43.7) |
|  | **2- & 3-FLUO (ng/L)** | 43.1 (26.4, 61.9) | 22.5 (8.3, 38.6) | 45.8 (25.3, 69.7) |
|  | **2-NAP (ng/L)** | 7.1 (-5.7, 21.7) | 12.6 (2.3, 24.0) | 25.3 (11.4, 41.0) |
|  | **1-PYR (ng/L)** | 51.5 (32.8, 72.7) | 14.9 (2.6, 28.7) | 39.2 (23.2, 57.2) |
|  | **Σ_4_OHPHEN (ng/L)** | 52.3 (30.4, 77.9) | 20.8 (4.8, 39.3) | 51.5 (32.2, 73.6) |
|  | **1-PHEN (ng/L)** | 36.6 (19.5, 56.1) | 13.8 (1.7, 27.2) | 33.9 (20.4, 48.8) |
|  | **2-PHEN (ng/L)** | 43.9 (23.5, 67.7) | 16.9 (1.9, 34.1) | 53.0 (32.6, 76.6) |
|  | **3-PHEN (ng/L)** | 50.8 (29.4, 75.6) | 18.3 (3.0, 35.8) | 42.9 (21.6, 68.1) |
|  | **4-PHEN (ng/L)** | 29.4 (11.8, 49.7) | 13.0 (-2.6, 31.0) | 26.6 (11.1, 44.2) |
| **8-OHdG (µg/L)** | **Σ_7_OHPAH (ng/L)** | 23.5 (12.1, 36.0) | 23.0 (11.3, 35.9) | 36.9 (25.0, 49.9) |
|  | **2- & 3-FLUO (ng/L)** | 33.9 (21.5, 47.6) | 33.8 (18.9, 50.6) | 55.2 (38.9, 73.4) |
|  | **2-NAP (ng/L)** | 21.4 (10.6, 33.2) | 19.9 (9.1, 31.8) | 33.9 (22.5, 46.4) |
|  | **1-PYR (ng/L)** | 48.6 (35.3, 63.2) | 24.1 (10.9, 38.8) | 40.6 (28.5, 53.8) |
|  | **Σ_4_OHPHEN (ng/L)** | 60.6 (45.0, 77.9) | 43.4 (25.0, 64.5) | 57.3 (42.5, 73.6) |
|  | **1-PHEN (ng/L)** | 44.8 (32.3, 58.5) | 29.2 (14.9, 45.2) | 35.3 (24.0, 47.7) |
|  | **2-PHEN (ng/L)** | 50.4 (35.2, 67.3) | 39.0 (21.6, 58.8) | 60.1 (44.4, 77.6) |
|  | **3-PHEN (ng/L)** | 49.3 (33.7, 66.8) | 36.5 (19.7, 55.7) | 50.5 (33.6, 69.4) |
|  | **4-PHEN (ng/L)** | 40.3 (26.5, 55.7) | 36.6 (18.4, 57.7) | 29.5 (16.7, 43.6) |
| a. Adjusted for maternal age, maternal race/ethnicity, maternal education, parity, pre-pregnancy BMI, season of sampling. Excluded pregnancy complications including gestational diabetes, gestational hypertension, and pre-eclampsia. | | | | |

**
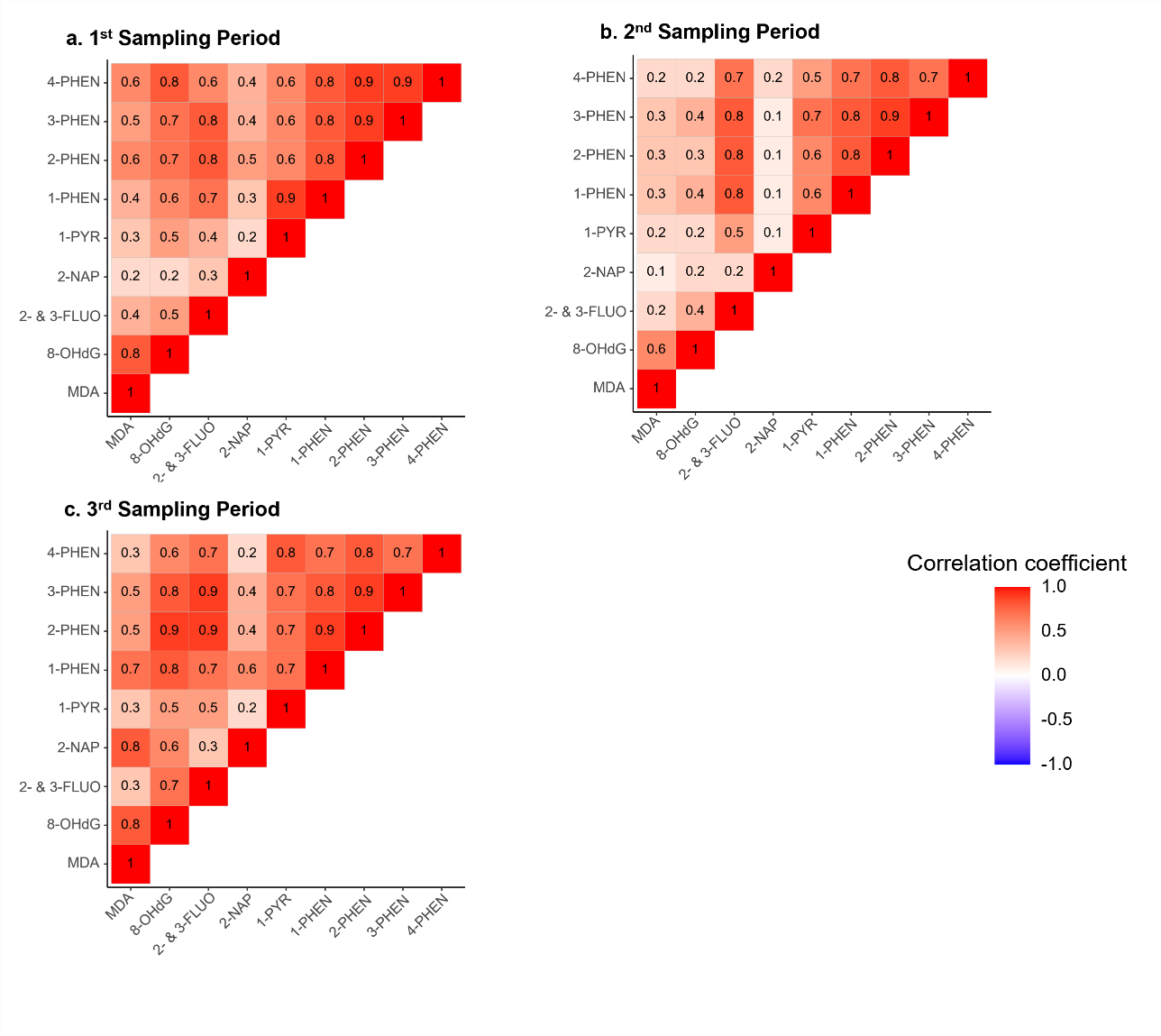
Figure S1 Pearson correlation coefficients of biomarker concentrations among study population**

**Figure S2 Linear regression for percentage changes of oxidative stress biomarker concentrations per doubling concentration of different PAH metabolites.** Adjusted for maternal age, maternal race/ethnicity, maternal education, parity, pre-pregnancy BMI, and sampling season.

**
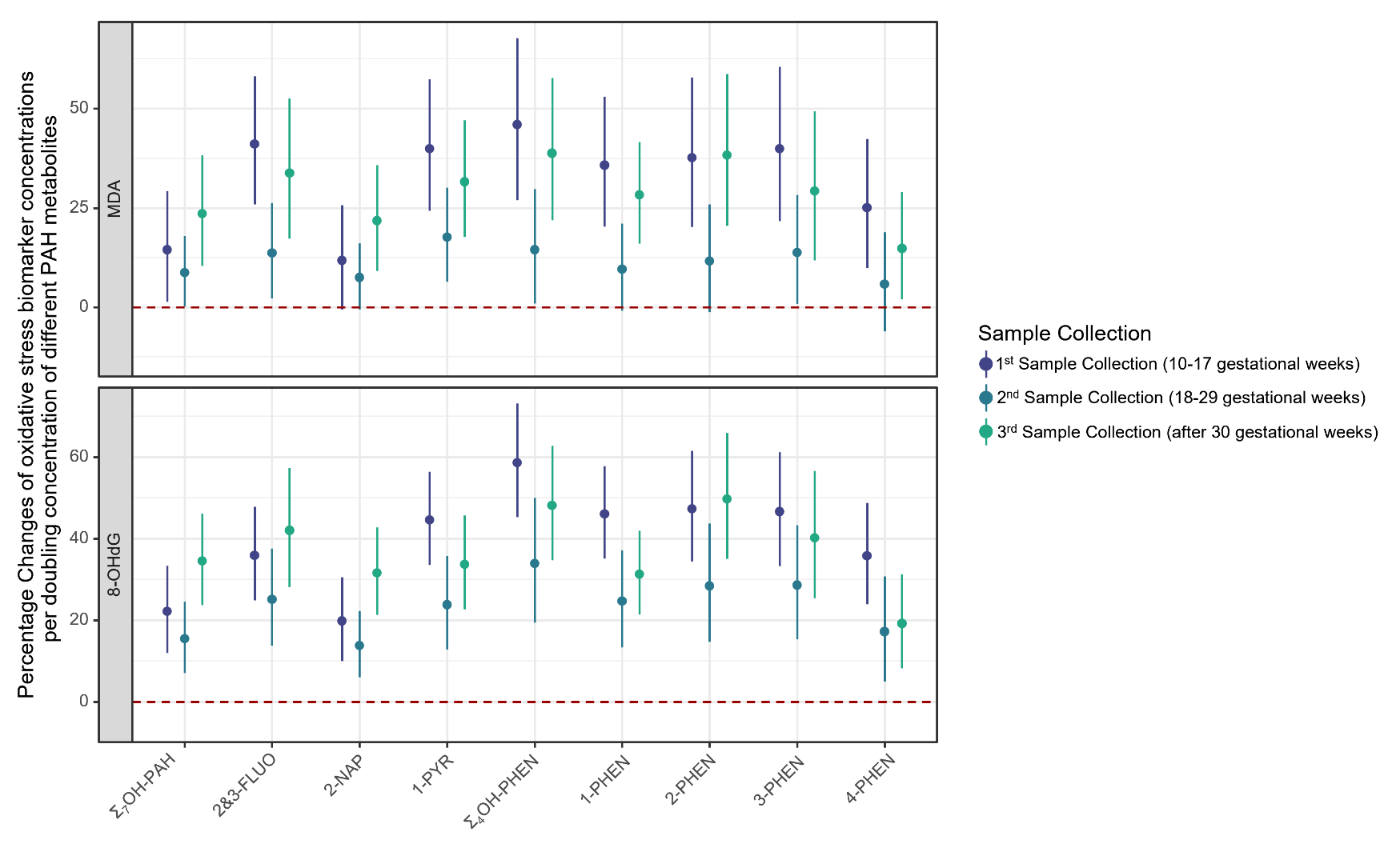
**

**Figure S3 Linear mixed regression models for percentage changes in oxidative stress biomarker concentrations per doubling concentration of different PAH metabolites, stratified by sampling season.** Adjusted for maternal age, maternal race/ethnicity, maternal education, parity, pre-pregnancy BMI.

**
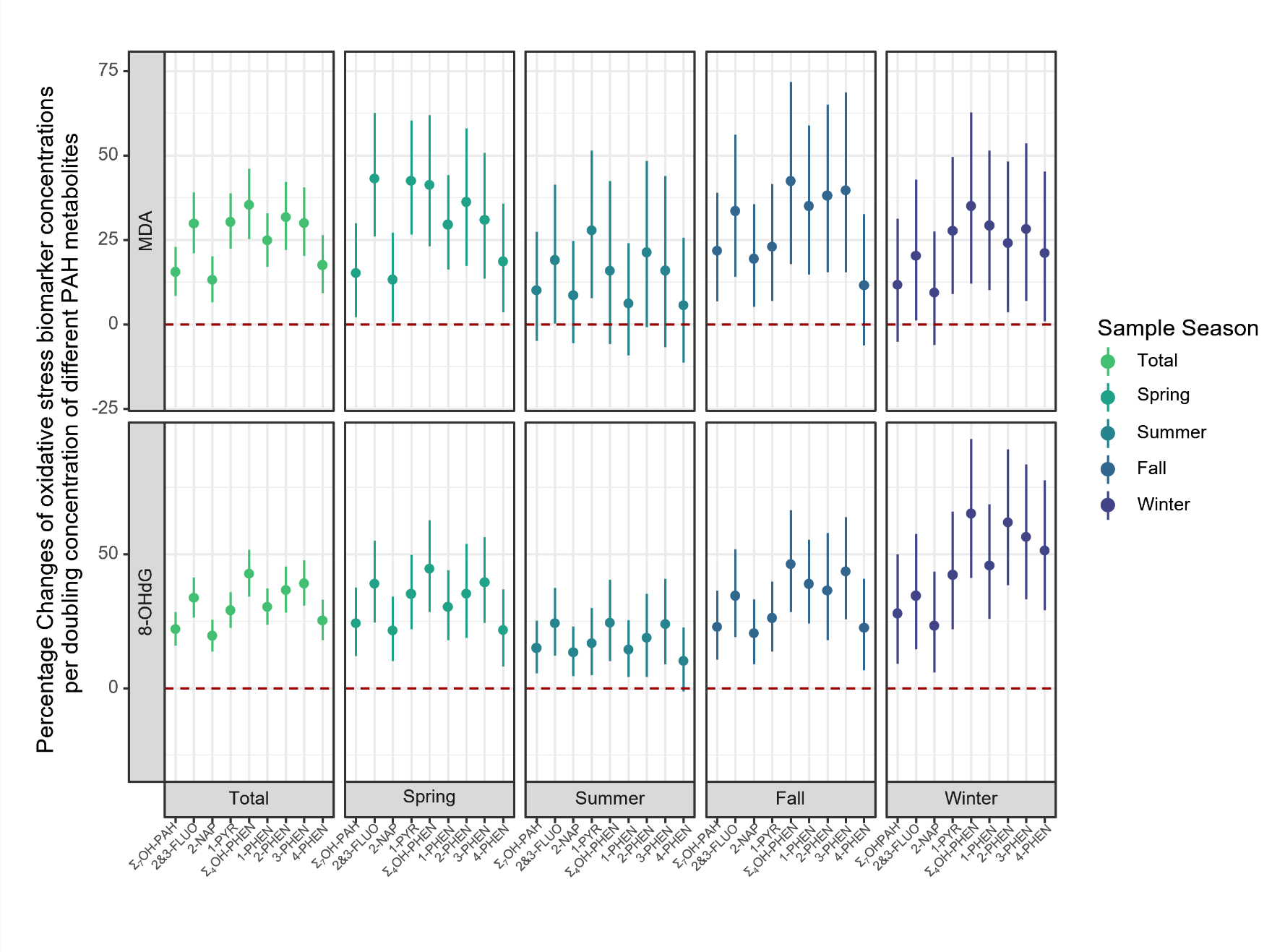
**

**Figure S4 Linear mixed regression models for percentage changes of oxidative stress biomarker concentrations per doubling concentration of different PAH metabolites, stratified by fetal sex.** Adjusted for maternal age, maternal race/ethnicity, maternal education, parity, pre-pregnancy BMI, and sampling season.

**
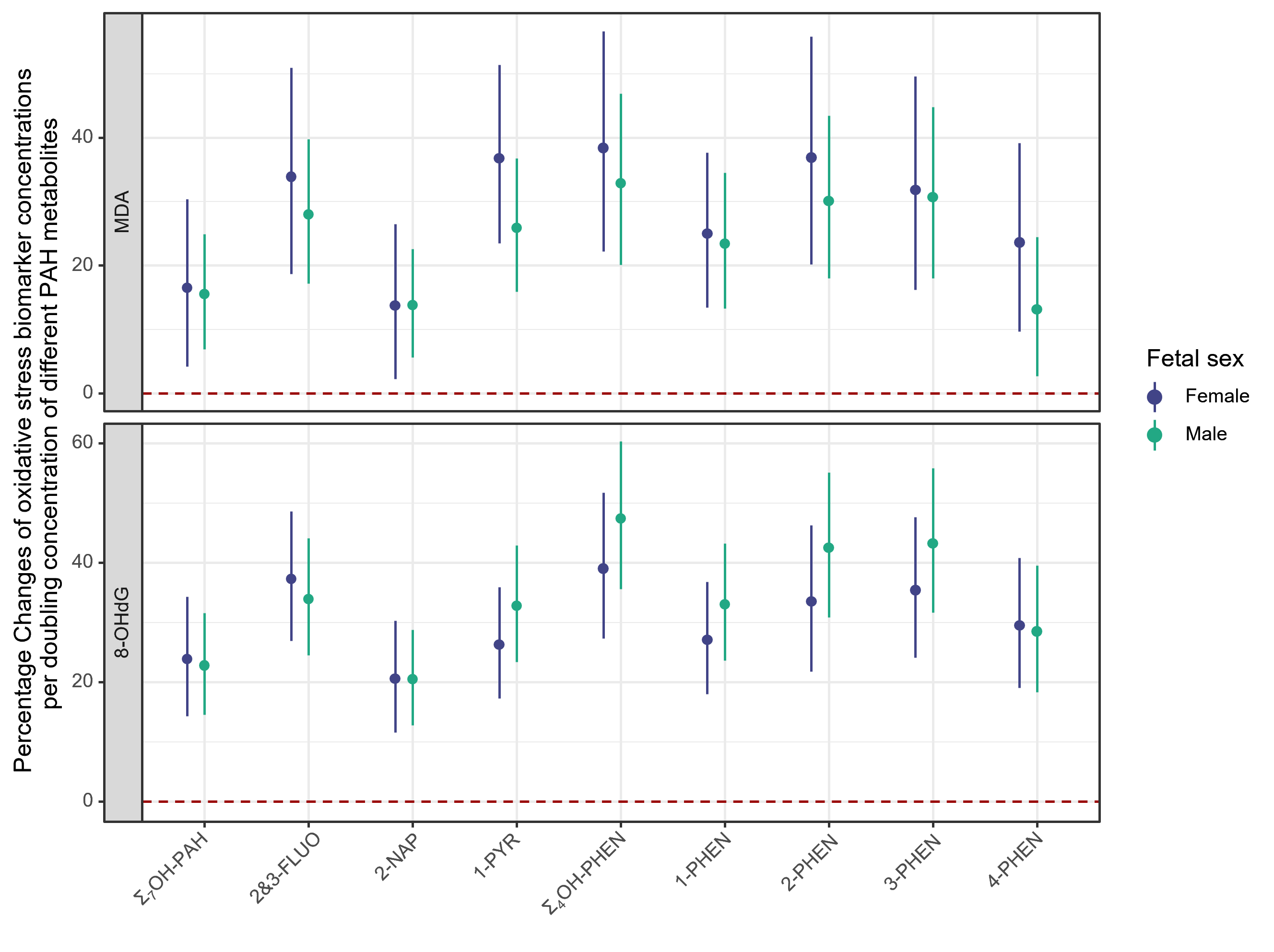
**

**Figure S5 Linear mixed regression models for percentage changes of oxidative stress biomarker concentrations per doubling concentration of different PAH metabolites, stratified by maternal race/ethnicity.** Adjusted for maternal age, maternal education, parity, pre-pregnancy BMI, and sampling season.


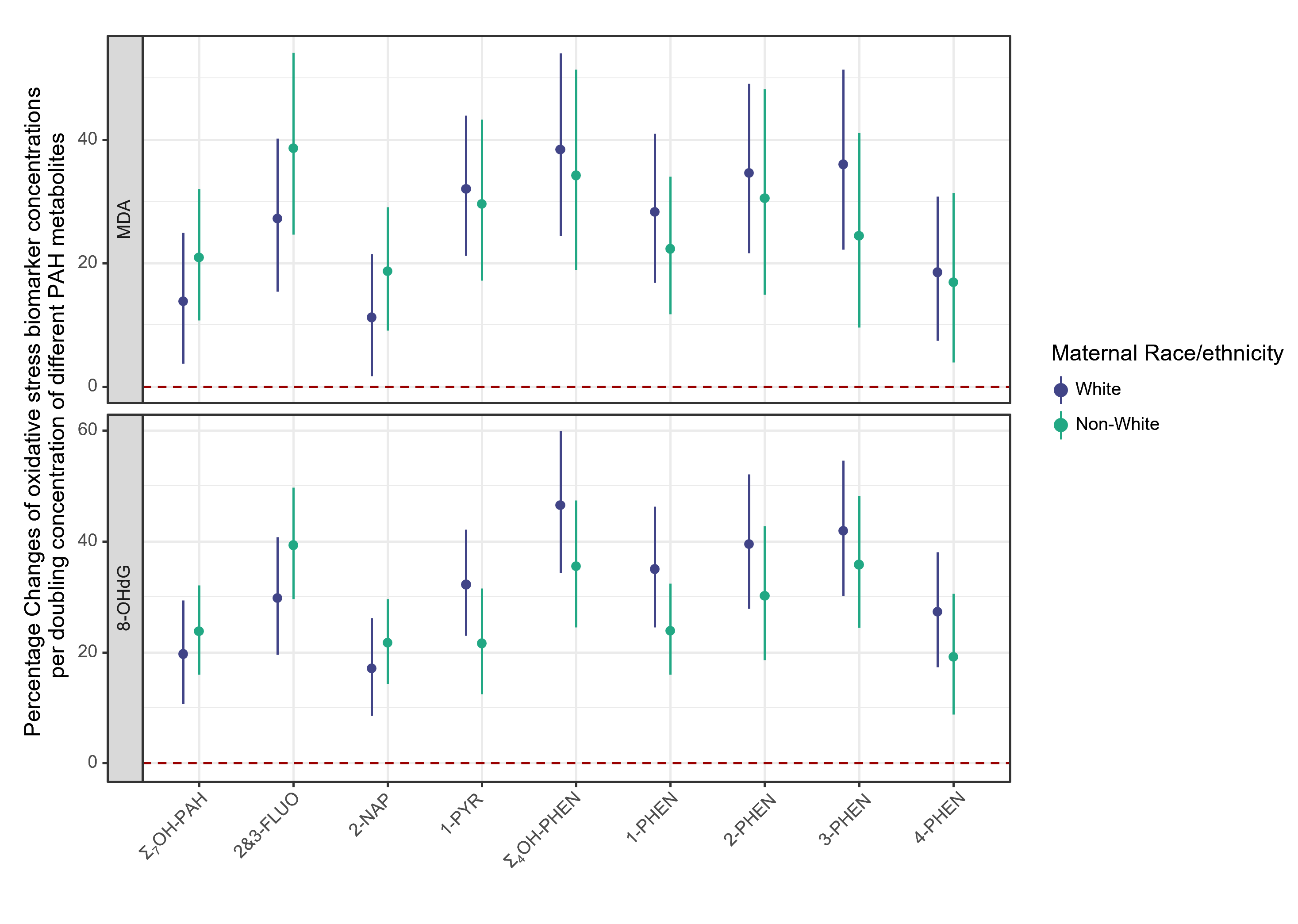

Supplement: Supplementary file 1 — Supplementary Material 1 [file 12940_2024_1107_MOESM1_ESM.docx]
